# Identifying the key genes and microRNAs in prostate cancer bone metastasis by bioinformatics analysis

**DOI:** 10.1002/2211-5463.12805

**Published:** 2020-03-19

**Authors:** Zhiguo Zhu, Yaoan Wen, Chunxiang Xuan, Qingping Chen, Qian Xiang, Jiamin Wang, Yangzhou Liu, Lianmin Luo, Shankun Zhao, Yihan Deng, Zhigang Zhao

**Affiliations:** ^1^ Department of Urology & Andrology Minimally Invasive Surgery Center Guangdong Provincial Key Laboratory of Urology The First Affiliated Hospital of Guangzhou Medical University Guangzhou China; ^2^ Department of Nursing Taian City Centre Hospital Branch Taian China; ^3^ School of Information Management Sun Yat‐Sen University Guangzhou China

**Keywords:** bioinformatics analysis, bone metastasis, differentially expressed miRNAs and genes, miRNA‐636, prostate adenocarcinoma

## Abstract

Prostate adenocarcinoma (PCa) is the most common cause of death due to malignancy among men, and bone metastasis is the leading cause of mortality in patients with PCa. Therefore, identifying the causes and molecular mechanism of bone metastasis is important for early detection, diagnosis and personalized therapy. In this study, we systematically analyzed molecular correlates of bone metastasis by bioinformatics analysis. A total of 12 differentially expressed microRNAs (miRNAs) and 102 differentially expressed genes were identified. Five miRNAs had prognostic significance in biochemical recurrence‐free survival (miR‐636, miR‐491‐5p, miR‐199b‐5p, miR‐199b‐3p, miR‐28‐3p). The differentially expressed genes were significantly enriched in extracellular matrix, cell‐substrate adhesion, collagen and integrin. Seven hub genes (*VCAN*, *COL3A1*, *COL1A1*, *APOE*, *COL1A2*, *SDC1*, *THY1*) with worse biochemical recurrence‐free survival and one hub gene (*MMP9*) with worse overall survival were detected. miR‐636, a novel oncogene, was found to be up‐regulated in bone metastatic PCa tissues and also predominately up‐regulated in human PCa cell lines. miR‐636 promoted cellular invasion and migration, and may promote bone metastasis via targeting *MBNL2*, *TNS1* and *STAB1*. In conclusion, we have successfully defined molecular signatures of bone metastasis in PCa.

## Graphical abstract

We systematically analyzed molecular correlates of prostate cancer bone metastasis by bioinformatics analysis. A total of 12 differentially expressed miRNAs and 102 differentially expressed genes were identified. A protein–protein interaction network and a miRNA–mRNA regulatory network were built to demonstrate their molecular correlates. miRNA‐636, a less studied miRNA, may promote bone metastasis via *MBNL2*, *TNS1* and *STAB1*.

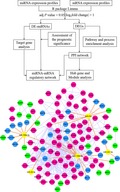

**Abbreviations**
BCR: biochemical recurrence
CI: confidence interval
DEG: differentially expressed gene
DE‐miRNA: differentially expressed miRNA
DFS: disease‐free survival
GEO: Gene Expression Omnibus
GO: gene ontology
HR: hazard ratio
MBNL2: muscleblind‐like splicing regulator 2
MCODE: Molecular Complex Detection
miRNA: microRNA
OS: overall survival
PCa: prostate adenocarcinoma
PPI: protein–protein interaction
STAB1: special AT‐rich sequence binding protein 1
TCGA: The Cancer Genome Atlas
TNS1: tensin 1

Prostate adenocarcinoma (PCa) is the most frequently diagnosed cancer in 105 countries (36 cancers in 185 countries) and the most common cause of death due to malignancy among men [1, 2]. Bone metastasis, the most common distant metastatic event of PCa, is the leading cause of mortality in patients with PCa. Therefore, identifying the causes and molecular mechanism of bone metastasis is important to early detection, diagnosis and personalized therapy.

MicroRNAs (miRNAs) are endogenous short (~22 nucleotides) RNAs that play important posttranscriptional regulatory roles by targeting mRNAs [3]. miRNAs promote the translational repression or degradation of mRNAs of protein‐coding genes by pairing the 3′‐UTRs of their targets [4]. Studies have suggested that miRNAs play a critical role in the initiation and progression of cancer, including bone metastasis [5, 6]. Bone metastasis involves complex interactions between tumor cells, bone cells and the microenvironment. miRNAs play a regulatory role in multiple steps in this process. Recent studies have carried out gene and miRNA expression profiling of metastatic tumor versus primary cancer for PCa [7, 8]. However, studies of molecular comparisons between primary PCa tissues and bone metastatic PCa tissues had not been found, which can potentially provide insights into the processes involved in the progression of bone metastasis.

Here, we systematically analyzed molecular correlates of bone metastasis using public datasets. The protein–protein interaction (PPI) network and miRNA–mRNA regulatory network were built to demonstrate the molecular correlates. In addition, we found that miRNA‐636, a less studied miRNA, perhaps promoted bone metastasis by muscleblind‐like splicing regulator 2 (*MBNL2*), tensin 1 (*TNS1*) and special AT‐rich sequence binding protein 1 (*STAB1*).

## Materials and methods

### Data collection

We compared genes and miRNAs expression between patients with primary PCa and patients with bone metastatic PCa. The gene expression profiles (http://www.ncbi.nlm.nih.gov/geo/query/acc.cgi?acc=GSE32269 and http://www.ncbi.nlm.nih.gov/geo/query/acc.cgi?acc=GSE77930) and the miRNA expression profile (http://www.ncbi.nlm.nih.gov/geo/query/acc.cgi?acc=GSE21036) were obtained from Gene Expression Omnibus (GEO) database (https://www.ncbi.nlm.nih.gov/geo/) [9, 10, 11]. We also collected serum exosomes from 15 patients with primary PCa and 15 patients with bone metastatic PCa for high‐throughput small RNA sequencing (S. H. Zhao, L. M. Luo, X. Qian, Z. G. Zhu, J. M. Wang, Y. Z. Liu, Y. H. Deng, J. T. Luo, R. Kang & Z. G. Zhao, unpublished data). In brief, total RNA was extracted from the collected serum exosomes, and small RNA libraries were established for sequencing by Illumina HiSeq 2500 (Illumina Inc.，San Diego, CA, USA) platform. Basic information of the included datasets was shown in Table 1. This study was approved by the Ethics Committee of The First Affiliated Hospital of Guangzhou Medical University. Informed written consent was obtained from the patients at the time of presentation for venous blood samples. Consent for publication was obtained from the participants. The study design conformed to the guidelines set by the Declaration of Helsinki.

**Table 1:** Basic information of included datasets. BM, bone metastasis; Exo, exosome; ID, identification; RNA‐seq, RNA sequencing.

ID	Platform	Data type	Author	Publication year	Country	Sample type	*n* (BM)	*n* (primary)
http://www.ncbi.nlm.nih.gov/geo/query/acc.cgi?acc=GSE21036	GPL8227	miRNA	Taylor BS [11]	2010	USA	Human tissues	3	99
http://www.ncbi.nlm.nih.gov/geo/query/acc.cgi?acc=GSE32269	GPL96	mRNA	Cai C [9]	2011	USA	Human tissues	29	22
http://www.ncbi.nlm.nih.gov/geo/query/acc.cgi?acc=GSE77930	GPL15695	mRNA	Kumar A [10]	2016	USA	Human tissues	20	14
Exo	RNA‐seq^a^	miRNA	Zhigang Zhao	S. H. Zhao, L. M. Luo, X. Qian, Z. G. Zhu, J. M. Wang, Y. Z. Liu, Y. H. Deng, J. T. Luo, R. Kang & Z. G. Zhao (Unpublished data)	China	Blood	15	15

^a^ Sequencing by Illumina HiSeq 2500 (Illumina Inc.) platform.

### Identification of differentially expressed miRNAs and differentially expressed genes

limma, an R package for the analysis of gene expression microarray data, was used to extract differentially expressed miRNAs (DE‐miRNAs) and differentially expressed genes (DEGs) [12]. The adjusted *P*‐value < 0.05 and |log_2_fold change| > 1 were chosen as the cutoff criteria.

### Pathway and process enrichment analysis of DEGs

Enrichment analysis for pathway and process among the DEGs was performed using Metascape (http://metascape.org) [13]. Metascape is a web‐based tool that combines functional enrichment, interactome analysis, gene annotation and membership search.

### PPI network, miRNA–mRNA regulatory network, hub gene and module analysis

Search Tool for the Retrieval of Interacting Genes and cytoscape software were applied to detect the interactions of the DEGs and construct the PPI network and miRNA–mRNA regulatory network [14, 15]. The cytoHubba plug‐in and Molecular Complex Detection (MCODE) plug‐in were used to identify hub genes and screen modules of the PPI network. All of the parameters of plug‐in were left as the defaults. The genes in modules and hub genes were also analyzed by Metascape.

### Assessment of the prognostic significance of DE‐miRNAs and target genes of miR‐636

Survival curves were plotted using the Kaplan–Meier method. The hazard ratio (HR) with 95% confidence intervals (CIs) and log rank *P*‐value were calculated to compare the biochemical recurrence free (BCR‐free) survival and overall survival (OS) of patients with PCa with different miRNA or mRNA expression using data from GEO: http://www.ncbi.nlm.nih.gov/geo/query/acc.cgi?acc=GSE21036. Gene Expression Profiling Interactive Analysis (GEPIA; http://gepia.cancer-pku.cn) was also used to assess the prognostic significance of target genes of miR‐636.

### Prediction of target genes of DE‐miRNAs

We used four target prediction databases, Target Scan [16], miRDB [17], miRPathDB [18] and miRWalk [19], to identify the target genes of DE‐miRNAs. The genes, predicted by four databases simultaneously, were selected as the objects for further research.

### Cell lines and cell culture

The human PCa cell line LNCaP and normal prostate epithelial cell line RWPE‐1 were obtained from Shanghai Chinese Academy of Sciences cell bank (China). Human PCa cell lines PC‐3M, 22Rv1 and C4‐2 were stored in our laboratory (Guangdong Provincial Key Laboratory of Urology). Human PCa cell lines PC‐3 and DU145 were presented by K. Weiting from Shandong University. RWPE‐1 was grown in keratinocyte serum‐free medium (Gibco, Shanghai, China) supplemented with 0.05 mg·mL^−1^ bovine pituitary extract and 5 ng·mL^−1^ human recombinant epidermal growth factor. LNCaP, PC‐3, PC‐3M and 22Rv1 were grown in RPMI‐1640 media (Gibco) supplemented with 10% fetal bovine serum (Gibco) and a 1% penicillin and streptomycin combination. C4‐2 and DU145 were grown in Dulbecco’s modified Eagle’s medium (Gibco) supplemented with 10% fetal bovine serum and 1% penicillin and streptomycin combination. All of the cells grew in standard cell culture conditions (atmosphere: air, 95%; carbon dioxide, 5%; temperature: 37 °C).

### Quantitative real‐time PCR

Total RNA from cells was isolated with TRIzol (Invitrogen; Thermo Fisher Scientific, Inc., Carlsbad, CA, USA) as described previously [20]. The primer and quantitative real‐time PCR detection kit of miRNA were purchased from FulenGen Co., Ltd. (Guangzhou, China). U6 was used as the endogenous controls. Relative quantification of miRNA levels was determined by the ^△△^ *C*_t_ method.

### Transient transfection

Inhibitor (Ribobio, Guangzhou, China) of miR‐636 and the negative control were used to knock down miR‐636. Cells were transfected with Lipofectamine 3000 (Invitrogen, Thermo Fisher Scientific, Inc.) according to the manufacturer’s protocol.

### Migration and invasion assays

Wound healing assays were conducted as described previously [21]. Cell migration was evaluated by measuring the difference in the wound area. Cell migration and invasion were also evaluated using a Transwell^®^ permeable support chamber (Corning Incorporated, Corning, NY, USA) with or without Matrigel (BD Biosciences, Franklin Lakes, NJ, USA) according to the manufacturer’s protocol.

### Statistical analysis

Data were analyzed using spss version 20.0 (SPSS Inc., Chicago, IL, USA) and graphpad prism version 8.0 (GraphPad Software, San Diego, CA, USA). A GraphPad Software*P*‐value < 0.05 indicated statistical significance.

## Results

**Fig. 1:**
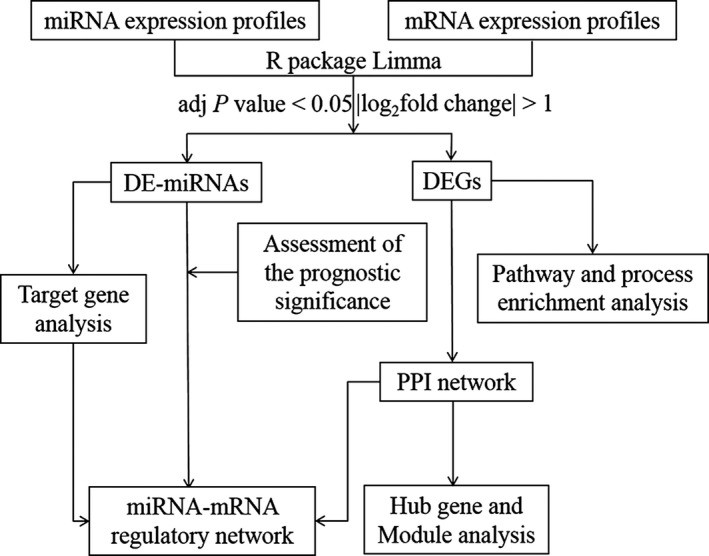
The flow chart of bioinformatics analysis. adj., adjusted.

The flow chart of bioinformatics analysis was presented in Fig. 1. First, we identified DE‐miRNAs and DEGs. Then we assessed the prognostic value of DE‐miRNAs to choose valuable DE‐miRNAs. We performed pathway and process enrichment analysis of DEGs, built a PPI network to describe the relationship of DEGs and performed Hub gene and Module analysis. We predicted target genes of selected DE‐miRNAs. Finally, we built the miRNA–mRNA regulatory network.

### Identification of DE‐miRNAs and DEGs

**Fig. 2:**
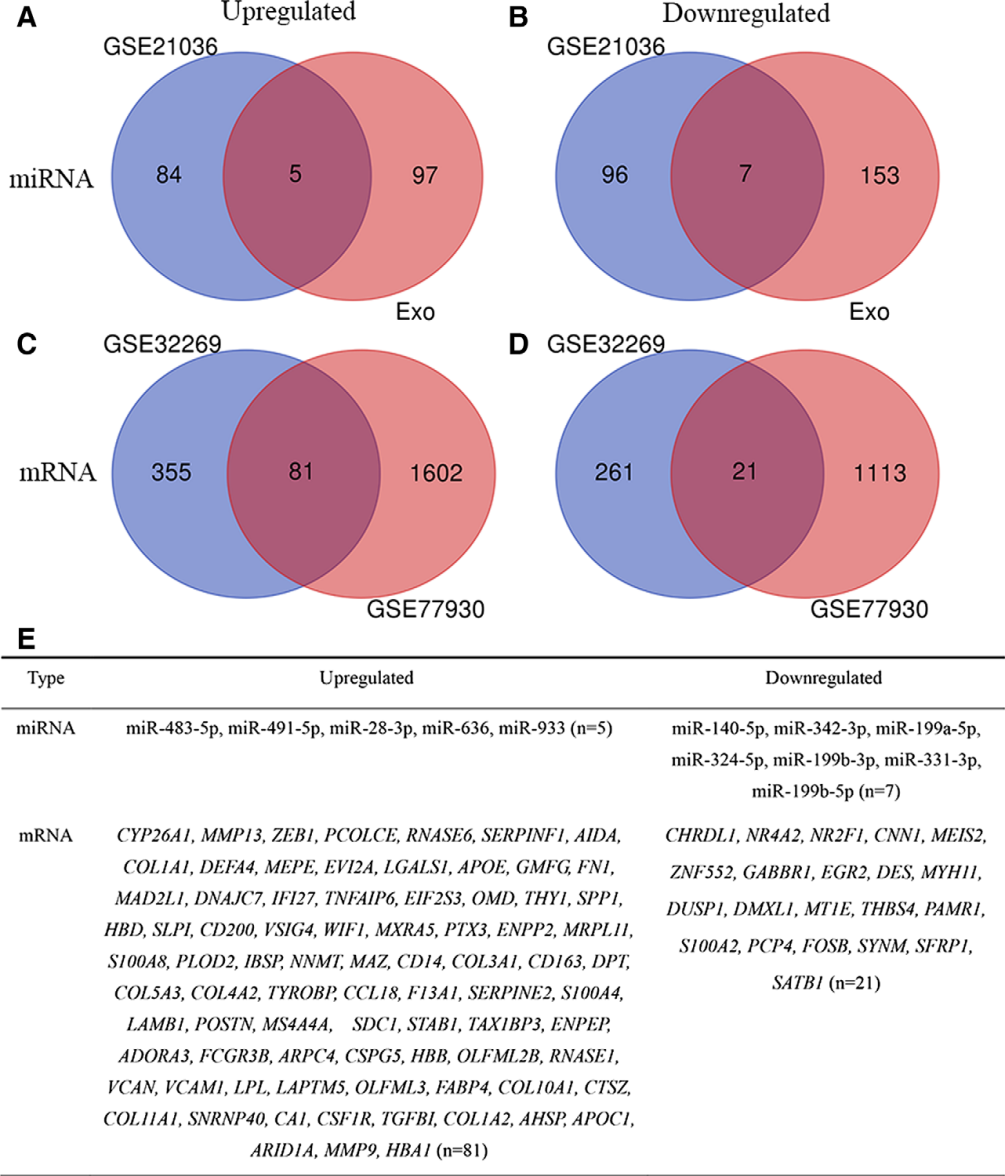
Identification of DE‐miRNAs and DEGs between bone metastatic PCa tissues and primary PCa tissues. (A, B) Venn diagrams for DE‐miRNAs. (C, D) Venn diagrams for DEGs. (E) List of DE‐miRNAs and DEGs.

**Fig. 3:**
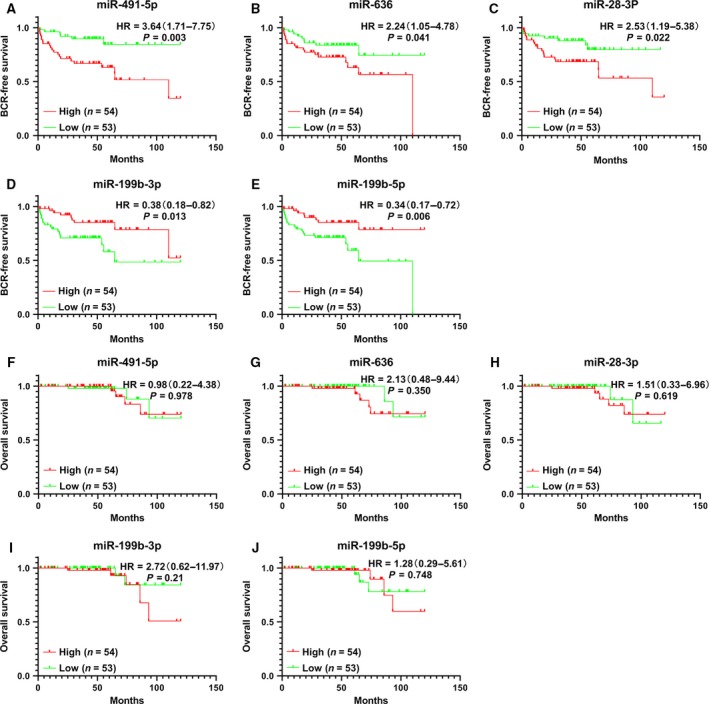
Prognostic significance of five DE‐miRNAs. (A–E) Kaplan–Meier survival curves about BCR. (F–J) Kaplan–Meier survival curves about death. This analysis was performed in GEO: http://www.ncbi.nlm.nih.gov/geo/query/acc.cgi?acc=GSE21036.

A total of 12 DE‐miRNAs (5 up‐regulated and 7 down‐regulated) and 102 DEGs (81 up‐regulated and 21 down‐regulated) were detected (Fig. 2). According to the results of log rank test, one miRNA had prognostic significance in OS (miR‐140‐5p), and five miRNAs had prognostic significance in BCR‐free survival (miR‐636, miR‐491‐5p, miR‐199b‐5p, miR‐199b‐3p and miR‐28‐3p) (Fig. 3; Figs S1 and S2). However, the change of miR‐140‐5p expression (down‐regulated) did not match its prognostic value in OS (HR, 7.08; 95% CI: 1.60–31.30; *P* = 0.03). Hence miR‐636, miR‐491‐5p, miR‐199b‐5p, miR‐199b‐3p and miR‐28‐3p were selected as the further research objects.

### Pathway and process enrichment analysis of DEGs

Metascape was used to perform pathway and process enrichment analysis. It integrates the mainstream database for enrichment analysis, including gene ontology (GO) processes, KEGG pathways, canonical pathways, reactome gene sets and CORUM complexes. The top 20 clusters with their representative enriched terms were shown in Table 2. The up‐regulated DEGs were primarily enriched in ‘extracellular structure organization’, ‘post‐translational protein phosphorylation’, ‘ossification’, ‘skeletal system development’, ‘endodermal cell differentiation’, ‘blood vessel development’, ‘leukocyte migration’, ‘cell‐substrate adhesion’, ‘signaling by PDGF’ and so on. The down‐regulated DEGs were primarily enriched in ‘naba matrisome associated’, ‘ossification’ and ‘response to steroid hormone’.

**Table 2:** The top 20 clusters with their representative enriched terms. 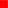, up‐regulated genes; 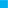, down‐regulated genes; KEGG, Kyoto Encyclopedia of Gene and Genome.

Gene list	Terms	Category	Description	Count	%	Log10(*P*)	Log10(*q*)
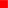	GO:0043062	GO Biological Processes	Extracellular structure organization	24	29.63	−22.45	−18.14
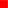	M5884	Canonical Pathways	naba core matrisome	20	24.69	−20.77	−16.76
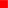	R‐HSA‐2173782	Reactome Gene Sets	Binding and uptake of ligands by scavenger receptors	9	11.11	−13.86	−10.5
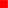 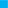	M5885	Canonical Pathways	naba matrisome associated	19	18.63	−9.47	−6.45
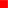	R‐HSA‐8957275	Reactome Gene Sets	Posttranslational protein phosphorylation	8	7.84	−7.72	−4.85
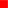	GO:0002274	GO Biological Processes	Myeloid leukocyte activation	14	17.28	−7.49	−4.66
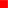 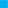	GO:0001503	GO Biological Processes	Ossification	12	11.76	−7.05	−4.24
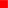	GO:0001501	GO Biological Processes	Skeletal system development	12	14.81	−6.81	−4.11
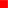	GO:0035987	GO Biological Processes	Endodermal cell differentiation	5	6.17	−6.39	−3.75
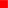	GO:0010035	GO Biological Processes	Response to inorganic substance	13	12.75	−6.15	−3.4
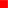	GO:0001568	GO Biological Processes	Blood vessel development	15	14.71	−6.11	−3.38
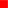	GO:0050900	GO Biological Processes	Leukocyte migration	12	11.76	−5.94	−3.27
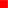	M118	Canonical Pathways	pid integrin A9B1 pathway	4	4.94	−5.85	−3.28
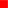	GO:0046503	GO Biological Processes	Glycerolipid catabolic process	5	6.17	−5.72	−3.16
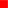	GO:0031589	GO Biological Processes	Cell‐substrate adhesion	10	9.8	−5.71	−3.09
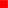	hsa05144	KEGG Pathway	Malaria	5	4.9	−5.7	−3.09
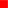	GO:0008285	GO Biological Processes	Negative regulation of cell proliferation	14	13.73	−5.37	−2.86
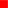	R‐HSA‐186797	Reactome Gene Sets	Signaling by PDGF (platelet derived growth factor)	5	4.9	−5.33	−2.83
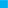	GO:0048545	GO Biological Processes	Response to steroid hormone	10	9.8	−5.2	−2.73
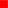	R‐HSA‐3560782	Reactome Gene Sets	Diseases associated with glycosaminoglycan metabolism	4	4.94	−4.97	−2.52

**Fig. 4:**
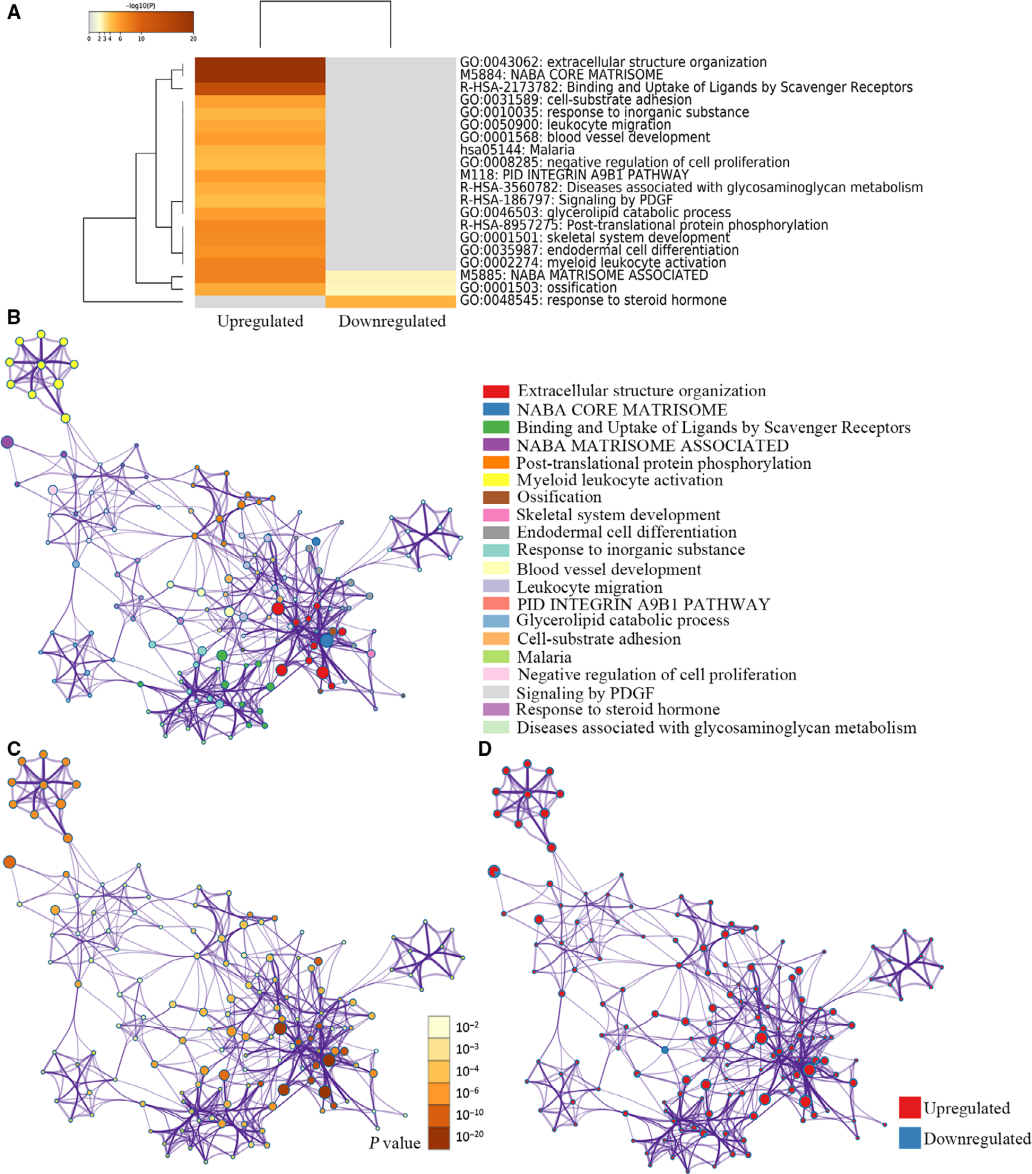
Visualizations of functional enrichment and interactome analysis results of DEGs. (A) Heatmap of enriched terms across input gene lists, colored by *P*‐values. (B–D) Network of enriched terms: (B) colored by enriched terms, (C) colored by *P*‐value, and (D) color‐coded based on the identities of the gene lists. PDGF, platelet derived growth factor.

These results were further visualized by Metascape. Figure 4A shows the Metascape enrichment analysis results as a clustered heatmap. In addition, an enrichment network was built to demonstrate the relationships between the terms. In the network, each node represented an enriched term and was colored by its cluster ID (Fig. 4B), *P*‐value (Fig. 4C) and the identities of the gene lists (Fig. 4D). The most notable enriched terms in the network were ‘extracellular structure organization’ and ‘naba core matrisome’, which were closely related to the metastasis of tumor cells.

### PPI network establishment and hub gene and module analysis for DEGs

**Fig. 5:**
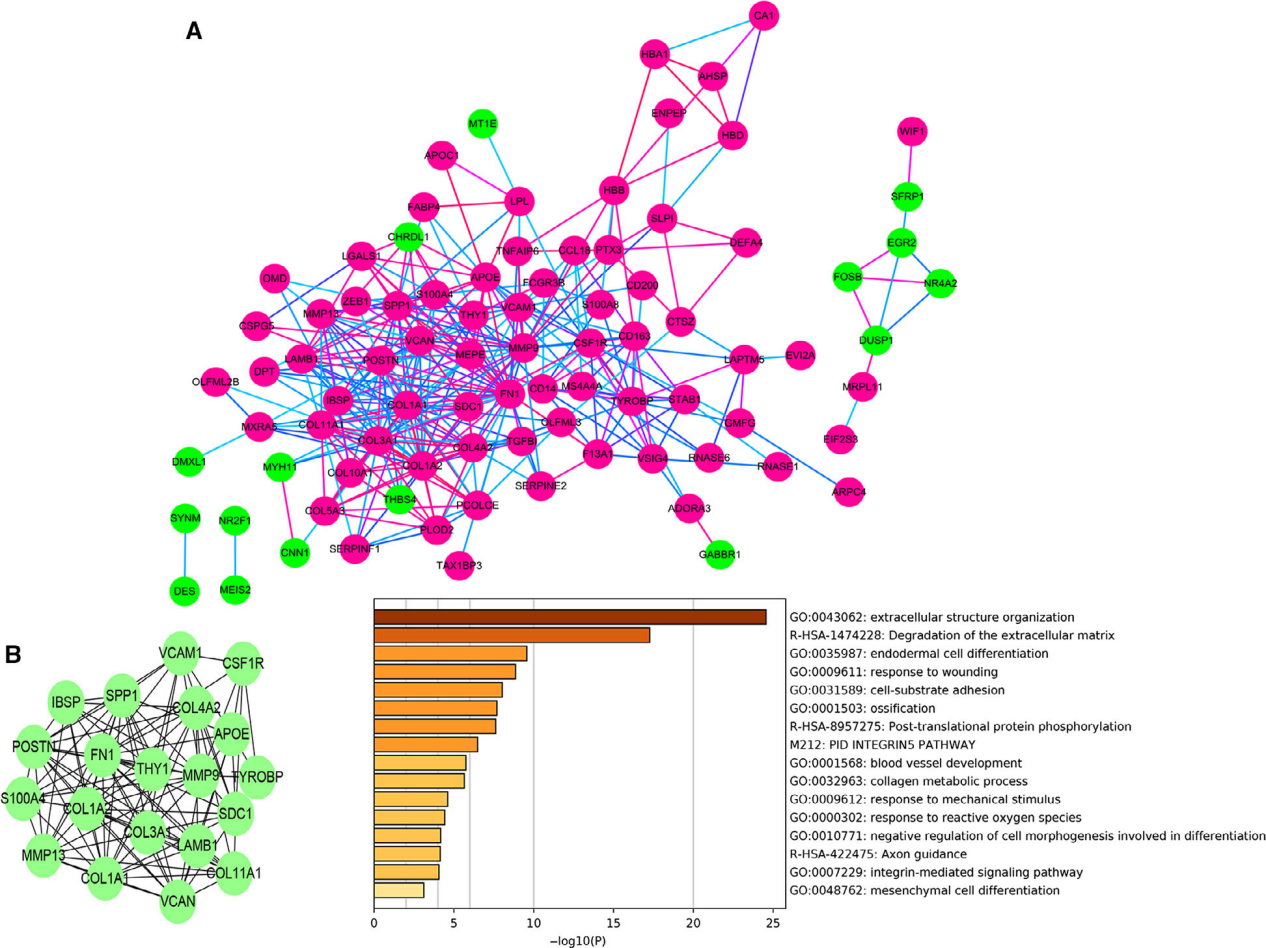
(A) PPI network of DEGs. Nodes were color‐coded based on the expression of DEGs (red, up‐regulated; green, down‐regulated). The edge color reflected the combined score of the genes (light blue → red = low score → high score). (B) Twenty hub genes with a higher degree of connectivity, and functional enrichment analysis of these genes.

**Fig. 6:**
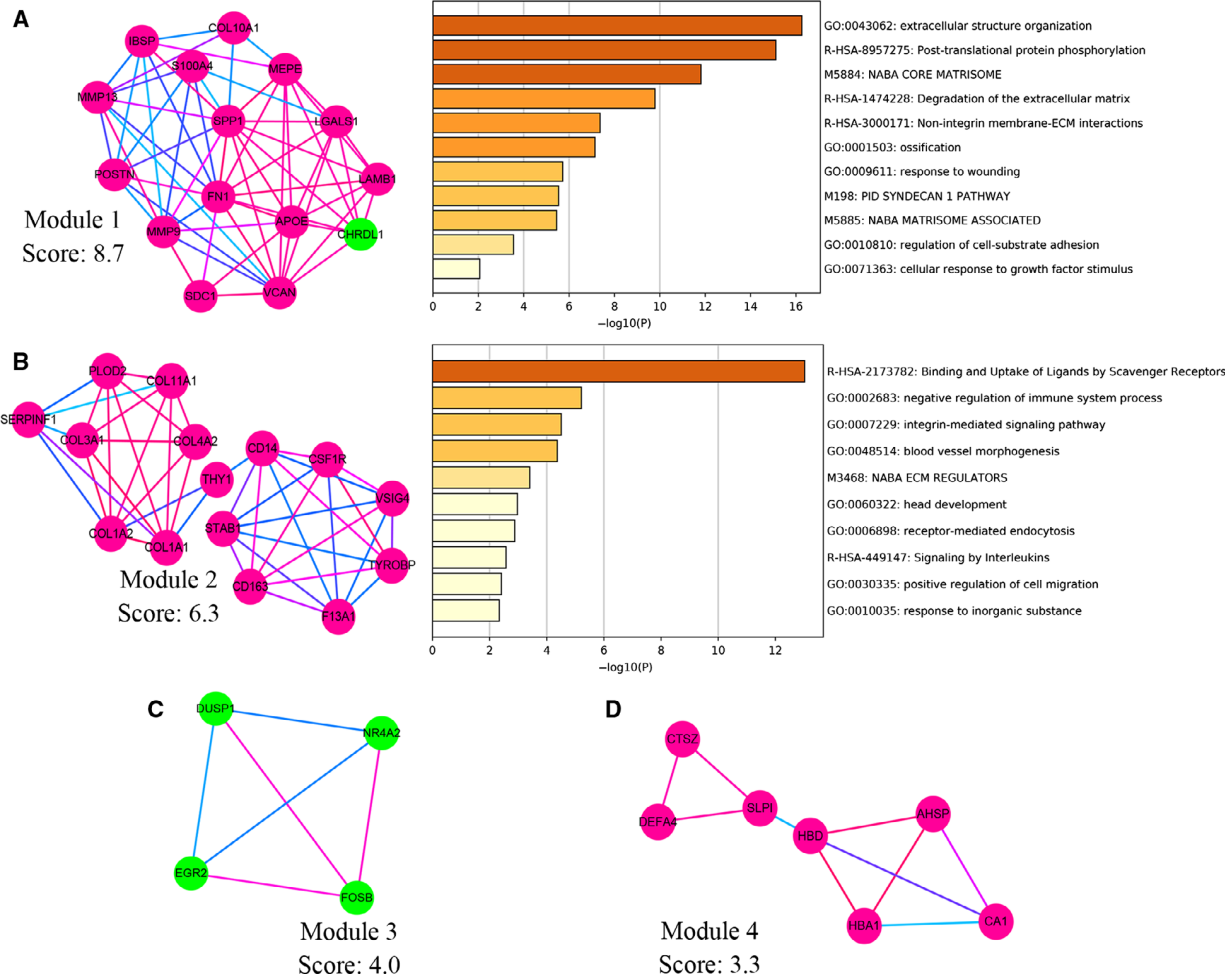
Module analysis of DEGs. Four modules screened from the PPI network. The genes of the top two modules were performed with functional enrichment analysis (A, B). (A–D) Nodes were color‐coded based on the expression of DEGs (red, up‐regulated; green, down‐regulated). The edge color reflected the combined score of the genes (light blue → red, low score → high score).

Based on the Search Tool for the Retrieval of Interacting Genes database, we built a PPI network of 102 DEGs, using cytoscape software (Fig. 5A). In the PPI network, nodes were color‐coded based on the expression of DEGs (red, up‐regulated; green, down‐regulated). The edge color reflected the combined score of the genes. The top 20 hub genes were detected using the 12 algorithms in cytoHubba plug‐in (Table S1). We selected 20 hub genes that were detected by more than six algorithms to build the hub gene PPI network (Fig. 5B). The 20 hub genes all had a higher degree of connectivity. These genes were enriched in a process related to bone metastasis: extracellular matrix, cell‐substrate adhesion, integrin, collagen and mesenchymal cell differentiation. Then we used MCODE plug‐in to identify significant modules in the DEGs PPI network. Four modules were detected (Fig. 6). We selected the top two modules, which had rich connections, to perform pathway and process enrichment analysis using Metascape. The enrichment analysis demonstrated that the modules were enriched in extracellular matrix, adhesion and cell migration.

### miRNA–mRNA regulatory network analysis

**Fig. 7:**
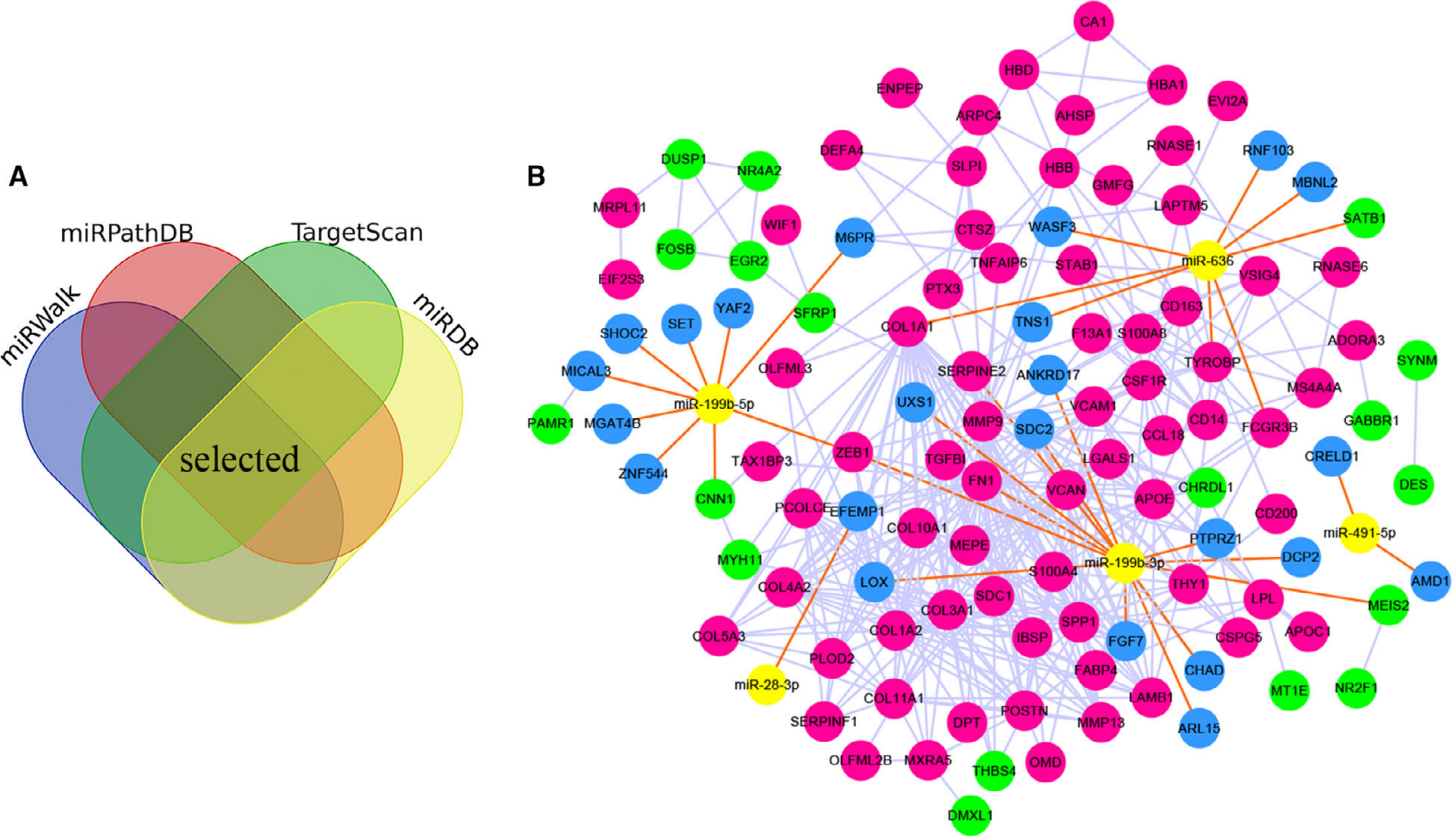
DE‐miRNAs–DEGs regulatory network. (A) A diagram illustrating prediction of target genes of miRNA. (B) miRNA–mRNA regulatory network. Nodes were color‐coded based on components (yellow, DE‐miRNAs; red, up‐regulated DEGs; green, down‐regulated DEGs; blue, target genes of DE‐miRNAs). The orange lines indicated the regulation relationship between DE‐miRNAs and its targets, whereas the silver lines indicated the relationship between DEGs.

We used four target prediction databases to identify the target genes of five DE‐miRNAs (Fig. 7A, Fig. S3). In total, 195 targets were identified, of which 32 targets had a different expression between patients with primary PCa and patients with bone metastatic PCa (adjusted *P*‐value < 0.05, not limited fold change; data are from GEO: http://www.ncbi.nlm.nih.gov/geo/query/acc.cgi?acc=GSE32269 and http://www.ncbi.nlm.nih.gov/geo/query/acc.cgi?acc=GSE77930). In addition, nine of the target genes (*ZEB1*, *SERPINE2*, *FN1*, *SATB1*, *TYROBP*, *CNN1*, *MEIS2*, *COL1A1* and *FCGR3B*) were DEGs. The contents of the miRNA–mRNA regulatory network include: (a) DE‐miRNAs (*n* = 5), (b) DEGs (adjusted *P*‐value < 0.05 and |log_2_fold change| > 1, *n* = 102), and (c) target genes (*n* = 36). In the network, miR‐199b‐5p, miR‐199b‐3p and miR‐636 had rich external connections (Fig. 7B). We used a webtool, OmicsBean, to find transcription factors in the network. Finally, we found two transcription factors (*ZNF552* and *ZNF544*) in the DE‐miRNAs–DEGs regulatory network.

### miR‐636 expression was up‐regulated in bone metastatic PCa tissues and promoted migration and invasion of PCa cells *in vitro*

**Fig. 8:**
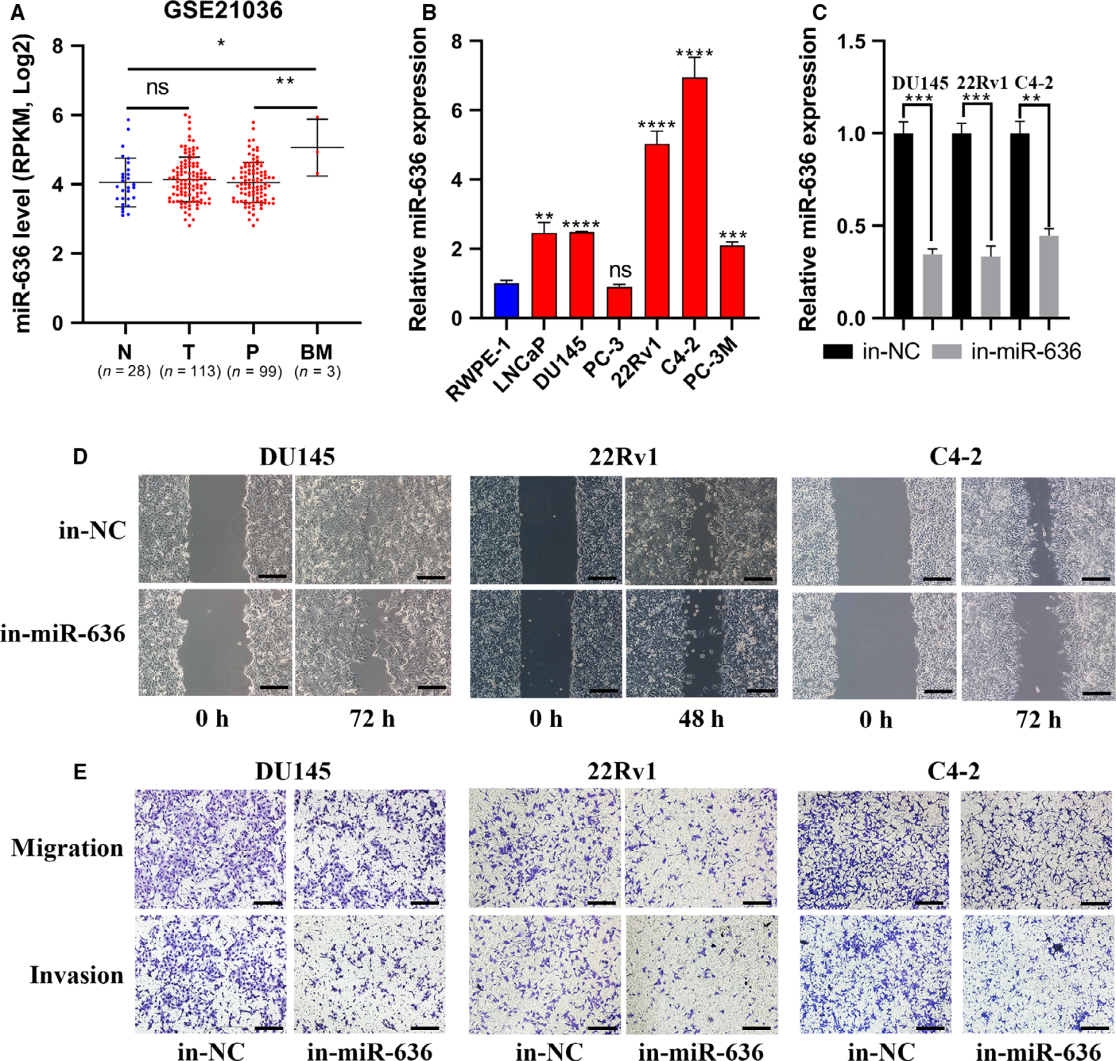
miR‐636 expression was elevated in bone metastatic PCa tissues and promoted PCa cell invasion and migration *in vitro*. (A) miR‐636 expression levels were increased in bone metastatic PCa tissues compared with that in primary tumor tissues by analyzing the PCa miRNA sequencing dataset from GEO: http://www.ncbi.nlm.nih.gov/geo/query/acc.cgi?acc=GSE21036; *n* values: numbers of biologically independent replicates. (B) miR‐636 was predominately up‐regulated compared with RWPE‐1, especially in metastatic, castration‐resistant 22Rv1 and C4‐2 PCa cell lines. (C) DU145, 22Rv1 and C4‐2 cell lines were respectively transfected with miR‐636 inhibitor to knock down miR‐636 expression. (D, E) The migration and invasion effect of miR‐636 was analyzed using Transwell and wound healing assays in DU145, 22Rv1 and C4‐2 cell lines. As shown, transfection of inhibitory miR‐636 can markedly impair the migrating and invading capacity. Scale bars: 400 μm (D, E). The error bars represent 95% CI (A) or standard deviation (B, C). ^ns^ *P *> 0.05; *P < 0.05; **P < 0.01; ***P < 0.001; ****P < 0.0001, Student’s *t*‐test. BM, bone metastatic PCa tissues, N, normal tissues; ns, no significance; P, primary tumor tissues; T, tumor tissues.

The role of the miR‐199 family has been widely investigated; however, the molecular mechanism of miR‐636 in tumor was still unclear. We analyzed the expression of miR‐636 in patients with PCa and prostate cell lines for the first time. As shown in Fig. 8A, miR‐636 level was elevated in the patients with bone metastatic PCa compared with that in patients with primary PCa. We further examined the expression levels of miR‐636 in normal prostate epithelial cell line RWPE‐1 and human PCa cell lines (LNCaP, PC‐3, PC‐3M,C4‐2, 22Rv1, DU145) and found that miR‐636 was predominately up‐regulated compared with RWPE‐1, especially in metastatic, castration‐resistant 22Rv1 and C4‐2 PCa cell lines (Fig. 8B). The migration and invasion effect of miR‐636 was analyzed using Transwell and wound healing assays in C4‐2, 22Rv1 and DU145 cells. As shown in Fig. 8D,E, transfection of inhibitory miR‐636 can markedly impair the migrating and invading capacity.

### *MBNL2*, *TNS1* and *STAB1* may be the targets of miR‐636 to promote bone metastasis

**Fig. 9:**
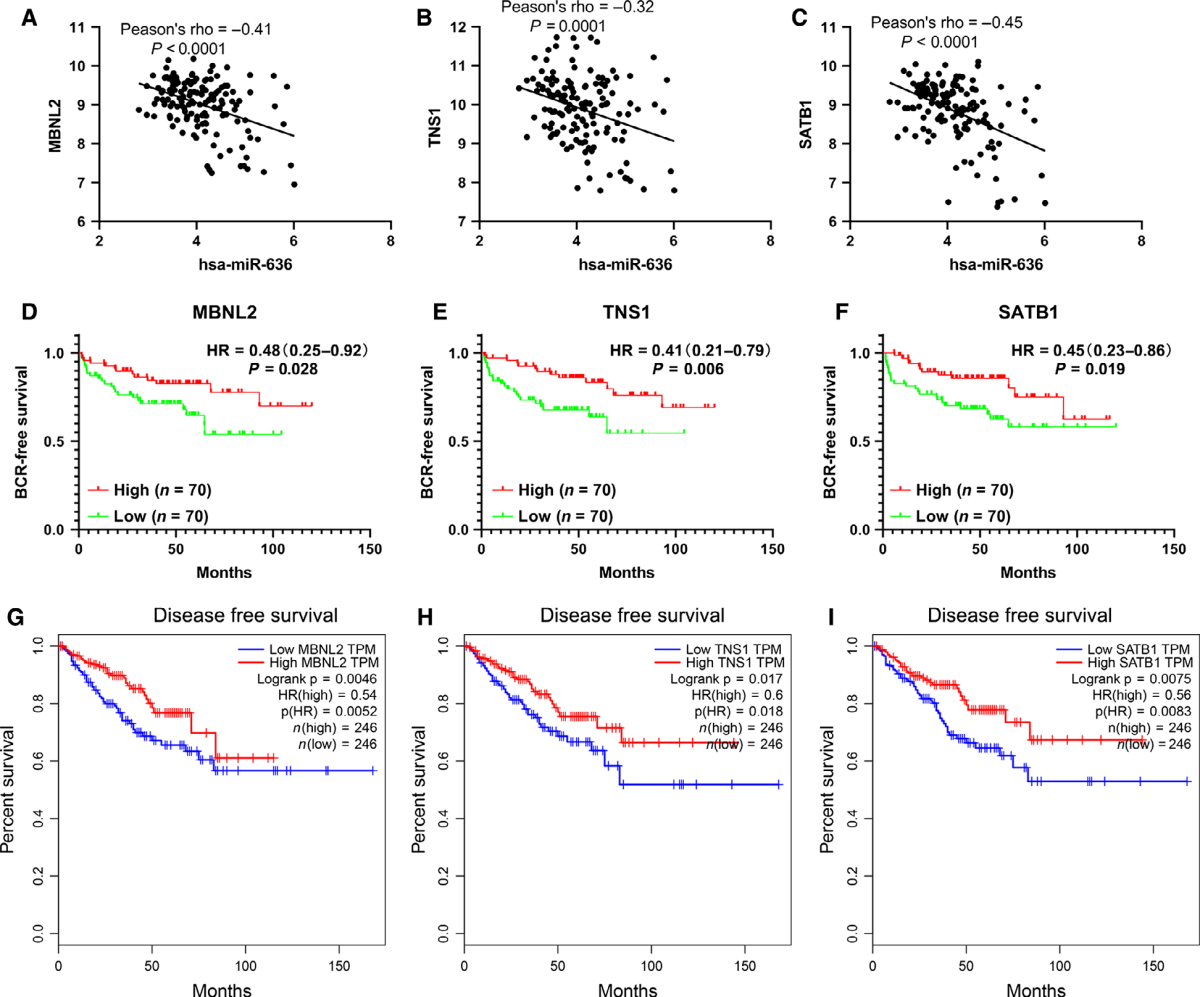
*MBNL2*, *TNS1* and *STAB1* may be the targets of miR‐636 to promote bone metastasis. (A–C) miR‐636 had significant negative correlations with *MBNL2* (Pearson’s rho = −0.41), *TNS1* (Pearson’s rho = −0.32) and *SATB1* (Pearson’s rho = −0.45) by analyzing the PCa miRNA sequencing dataset and mRNA sequencing dataset from GEO: http://www.ncbi.nlm.nih.gov/geo/query/acc.cgi?acc=GSE21036. (D–F) *MBNL2*, *TNS1* and *STAB1* had better BCR‐free survival in patients with PCa (data from GEO: http://www.ncbi.nlm.nih.gov/geo/query/acc.cgi?acc=GSE21036). (G–I) *MBNL2*, *TNS1* and *STAB1* had better DFS in patients with PCa (data are from TCGA).

In the miRNA–mRNA regulatory network, miR‐636 had eight external connections: *MBNL2*, *RNF103*, *TNS1*, *WASF3*, *SATB1*, *TYROBP*, *COL1A1* and *FCGR3B*. We identified the relationship between miR‐636 expression and target gene expression in GEO: http://www.ncbi.nlm.nih.gov/geo/query/acc.cgi?acc=GSE21036. miR‐636 had significant negative correlations with *MBNL2* (Pearson’s rho = −0.41), *TNS1* (Pearson’s rho = −0.32) and *SATB1* (Pearson’s rho = −0.45) (Fig. 9A–C). Figure S4A–F showed their expression was elevated in bone metastatic PCa tissues. *MBNL2* and *TNS1* levels were also elevated in PCa tissues compared with that in adjacent normal tissues [data are from The Cancer Genome Atlas (TCGA); Fig. S4G–I]. Furthermore, *MBNL2*, *SATB1* and *TNS1* had prognostic significance in BCR‐free survival (GEO: http://www.ncbi.nlm.nih.gov/geo/query/acc.cgi?acc=GSE21036; Fig. 9D–F) and disease‐free survival (DFS; TCGA; Fig. 9G–I), but not in OS.

## Discussion

PCa is the second most common cancer and the most common cause of death due to malignancy among men [2]. With the widespread adoption of the prostate‐specific antigen screening, an increasing number of people are diagnosed at an early stage of the disease. Due to the uneven distribution of medical resources, many patients are still diagnosed with PCa with distant metastasis, including bone metastasis. Bone metastasis is the leading cause of mortality in patients with PCa. Hence it is important to identify the molecular mechanism of bone metastasis.

In this study, we systematically analyzed molecular correlates of bone metastasis for the first time, using public databases (GEO: http://www.ncbi.nlm.nih.gov/geo/query/acc.cgi?acc=GSE32269, http://www.ncbi.nlm.nih.gov/geo/query/acc.cgi?acc=GSE77930 and http://www.ncbi.nlm.nih.gov/geo/query/acc.cgi?acc=GSE21036) and our small RNA sequencing data. Through bioinformatics analysis, 5 DE‐miRNAs and 102 DEGs were identified. To further explore the relationships between them, we performed pathway and process enrichment analysis, built a PPI network and miRNA–mRNA regulatory network, and did hub gene and module analysis.

In the PPI network, we found that there were rich interactions between the DEGs. The results of Metascape demonstrated that the 102 DEGs were enriched in extracellular structure organization, cell‐substrate adhesion, integrin, endodermal cell differentiation and ossification. In order to identify the key genes, we further performed hub gene analysis and module analysis. We selected 20 hub genes by 12 algorithms in cytoHubba plug‐in. Seven hub genes (*VCAN*, *COL3A1*, *COL1A1*, *APOE*, *COL1A2*, *SDC1* and *THY1*) with worse BCR‐free survival and one hub gene (*MMP9*) with worse OS were detected. The 20 hub genes were enriched in extracellular matrix, cell‐substrate adhesion, integrin, collagen and mesenchymal cell differentiation, which played a vital role in bone metastasis [22, 23, 24, 25, 26]. MCODE can find molecular complexes in the PPI network. We found four modules by MCODE. The top two modules were selected to perform pathway and process enrichment analysis. The enrichment analysis demonstrated that the modules were enriched in extracellular matrix, adhesion and cell migration, which were similar to the results of hub genes. Nineteen genes (*MMP13*, *THY1*, *CSF1R*, *TYROBP*, *SPP1*, *IBSP*, *COL1A1*, *COL4A2*, *POSTN*, *APOE*, *MMP9*, *S100A4*, *COL1A2*, *COL3A1*, *VCAN*, *COL11A1*, *SDC1*, *LAMB1* and *FN1*) were detected by hub gene analysis and module analysis simultaneously. Therefore, we hypothesized that these hub genes played an important role in bone metastasis of PCa.

We identified five DE‐miRNAs by bioinformatics analysis: miR‐636, miR‐491‐5p, miR‐199b‐5p, miR‐199b‐3p and miR‐28‐3p. Three miRNAs (miR‐636, miR‐491‐5p and miR‐28‐3p) had worse BCR‐free survival, and two miRNAs (miR‐199b‐5p and miR‐199b‐3p) had better BCR‐free survival. Four target prediction databases were used to identify the targets of DE‐miRNAs. Then, we built the miRNA–mRNA regulatory network. miR‐491‐5p and miR‐28‐3p have few external connections in Fig. 7. In addition, studies have reported that miR‐491‐5p was a tumor suppressor in a variety of tumors, including prostate cancer [27, 28, 29, 30]. This was inconsistent with our findings. miR‐491‐5p may play different roles at different stages of the disease. miR‐28‐3p and miR‐28‐5p were two different mature miRNA sequences that were excised from opposite arms of the stem‐loop pre‐miR‐28. miRNA‐28‐5p played the role of tumor suppressor in glioma, melanoma, pancreatic cancer, liver cancer, colorectal cancer and prostate cancer [31, 32, 33, 34, 35, 36]. However, miR‐28‐3p facilitated tumor progression in colorectal cancer and nasopharyngeal cancer [37, 38]. In the network, miR‐199b‐5p, miR‐199b‐3p and miR‐636 had rich external connections. Studies had reported that the miR‐199 family, as a tumor suppressor, inhibited tumor growth and metastasis [39, 40, 41, 42, 43]. Our group also found that miR‐199b‐5p suppressed cell proliferation, migration and invasion by down‐regulating *DDR1* in PCa (S. H. Zhao, L. M. Luo, X. Qian, Z. G. Zhu, J. M. Wang, Y. Z. Liu, Y. H. Deng, J. T. Luo, R. Kang & Z. G. Zhao, unpublished data).

Until now, the research on the role of miR‐636 in tumors was lacking. Studies demonstrated that miR‐636 was up‐regulated in SW620 cell (lymph node metastatic derivatives, human colon adenocarcinoma cells) and pancreatic carcinoma [44, 45]. High miR‐636 expression was associated with BCR after radical prostatectomy in PCa [46]. However, there was also a report that miR‐636 might function as a tumor suppressor in hepatocellular carcinoma [47]. We found that miR‐636 level was elevated in bone metastatic PCa tissues and also predominately up‐regulated in human PCa cell lines, especially in metastatic, castration‐resistant 22Rv1 and C4‐2 PCa cell lines. We evaluated the migration and invasion effect of miR‐636 in high‐expression cell lines (C4‐2, 22Rv1) and a relatively low‐expression cell line (DU145). Knocking down miR‐636 can markedly impair the migrating and invading capacity. In addition, miR‐636 had worse BCR‐free survival (HR, 2.24; 95% CI, 1.05–4.78; *P* = 0.04). *MBNL2*, *TNS1* and *STAB1* may be the targets of miR‐636 to promote bone metastasis. The mechanism of these genes in prostate cancer had not been reported. *MBNL2* repressed embryonic stem cell‐specific alternative splicing and reprogramming [48]. A study reported that *MBNL2* was a tumor suppressor in hepatocarcinogenesis [49]. *TNS1* was a focal adhesion molecule. Hic‐5 (TGFβ1i1) could control tumor extracellular matrix remodeling through interaction with TNS1 [50]. Most studies suggested that *STAB1* was an oncogene and up‐regulated in many cancers [51]. *STAB1* was involved in tumor immunity, epithelial‐to‐mesenchymal transition, metastasis and multidrug resistance. *MBNL2*, *TNS1* and *STAB1* were all reduced in the patients with bone metastatic PCa compared with that in patients with primary PCa (GEO: http://www.ncbi.nlm.nih.gov/geo/query/acc.cgi?acc=GSE32269 and http://www.ncbi.nlm.nih.gov/geo/query/acc.cgi?acc=GSE77930). According to the results of Gene Expression Profiling Interactive Analysis, *MBNL2* and *TNS1* levels were differentially down‐regulated in PCa tissues compared with that in the adjacent normal tissues (Fig. S4G,H). The expression of *STAB1* was not different between PCa tissues and the adjacent normal tissues (Fig. S4I). In addition, *MBNL2*, *TNS1* and *STAB1* had better BCR‐free survival and DFS in PCa. We will further explore the roles of miR‐636 and its targets in PCa bone metastasis.

In conclusion, we successfully defined molecular signatures of bone metastasis in PCa. The identified DE‐miRNAs and DEGs might play a key role in PCa bone metastasis. miRNA‐636, a novel oncogene, might promote bone metastasis via targeting *MBNL2*, *TNS1* and *STAB1*.

## Conflict of interest

The authors declare no conflict of interest.

## Author contributions

Z Zhu designed the study, analyzed data and wrote the manuscript. Z Zhao designed the study and wrote the manuscript. YW, CX and QC analyzed the data and reviewed the paper. QX, JW and YL provided ideas, collected data and reviewed the paper. LL, SZ and YD provided recommendations and reviewed the paper. All authors have read and approved the final manuscript.

## Supporting information


**Fig. S1.** Prognostic significance of seven DE‐miRNAs for BCR. This analysis was performed in GEO: GSE21036.
**Fig. S2.** Prognostic significance of seven DE‐miRNAs for death. This analysis was performed in GEO: GSE21036.
**Fig. S3.** Identification of target genes of miRNA using four target prediction databases.
**Fig. S4.** *MBNL2*, *TNS1* and *STAB1* expression were elevated in bone metastatic PCa tissues. (A–C) The expression of* MBNL2*, *TNS1* and *STAB1* in GEO: GSE32269. (D–F) The expression of* MBNL2*, *TNS1* and *STAB1* in GEO: GSE77930. (G–I) *MBNL2* and *TNS1* levels were elevated in PCa tissues compared with that in adjacent normal tissues (data are from TCGA). ^ns^ *P *> 0.05; *P < 0.05; **P < 0.01; ***P < 0.001; ****P < 0.0001, Student’s *t*‐test. BM, bone metastatic tissues; N, adjacent normal tissues; ns, no significance; T, tumor tissues.
**Table S1.** The top 20 hub genes detected using the 12 algorithms in cytoHubba plug‐in.
