# Supplementary material for: Identifying the key genes and microRNAs in prostate cancer bone metastasis by bioinformatics analysis
Source: FEBS Open Bio. 2020 Mar 19;10(4):674–88. doi: 10.1002/2211-5463.12805 (PMC7137804; doi:10.1002/2211-5463.12805)
Supplement: Supplementary file 1 — Fig. S1. Prognostic significance of seven DE‐miRNAs for BCR. This analysis was performed in GEO: GSE21036. Fig. S2. Prognostic significance of seven DE‐miRNAs for death. This analysis was performed in GEO: GSE21036. Fig. S3. Identification of target genes of miRNA using four target prediction databases. Fig. S4. MBNL2, TNS1 and STAB1 expression were elevated in bone metastatic PCa tissues. (A–C) The expression of MBNL2, TNS1 and STAB1 in GEO: GSE32269. (D–F) The expression of MBNL2, TNS1 and STAB1 in GEO: GSE77930. (G–I) MBNL2 and TNS1 levels were elevated in PCa tissues compared with that in adjacent normal tissues (data are from TCGA). ns P > 0.05; *P < 0.05; **P < 0.01; ***P < 0.001; ****P < 0.0001, Student’s t‐test. BM, bone metastatic tissues; N, adjacent normal tissues; ns, no significance; T, tumor tissues. Table S1. The top 20 hub genes detected using the 12 algorithms in cytoHubba plug‐in. [file FEB4-10-674-s001.pdf]

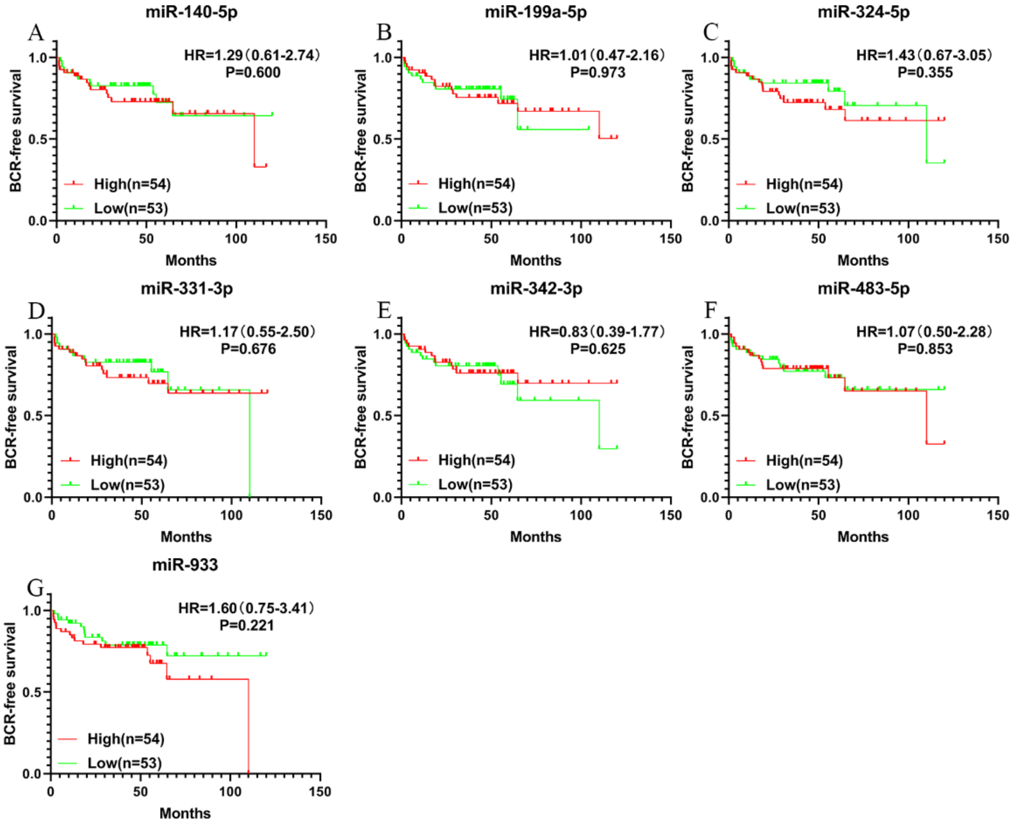

Fig. S1. Prognostic significance of seven DE-miRNAs for BCR.

This analysis was performed in GEO: GSE21036.

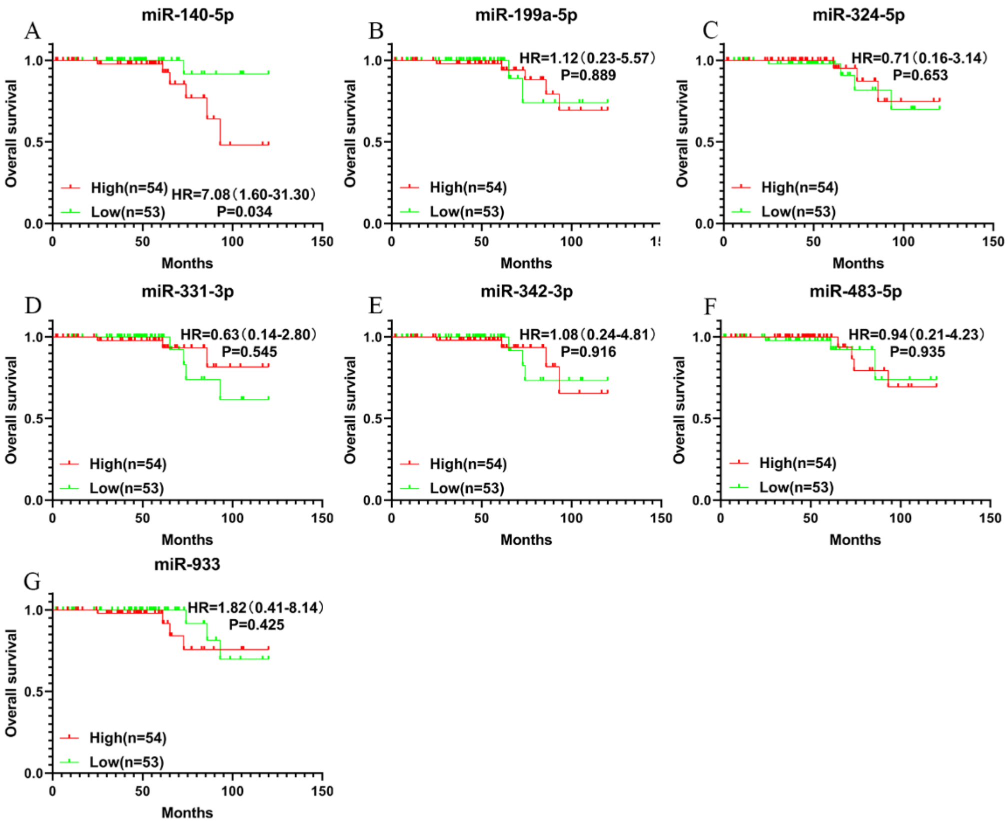

Fig. S2. Prognostic significance of seven DE-miRNAs for death.

This analysis was performed in GEO: GSE21036.

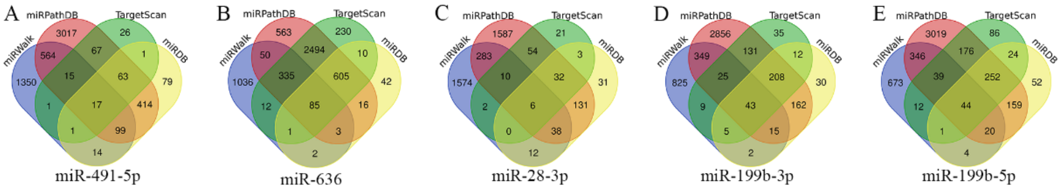

Fig. S3. Identification of target genes of miRNA using four target prediction databases.

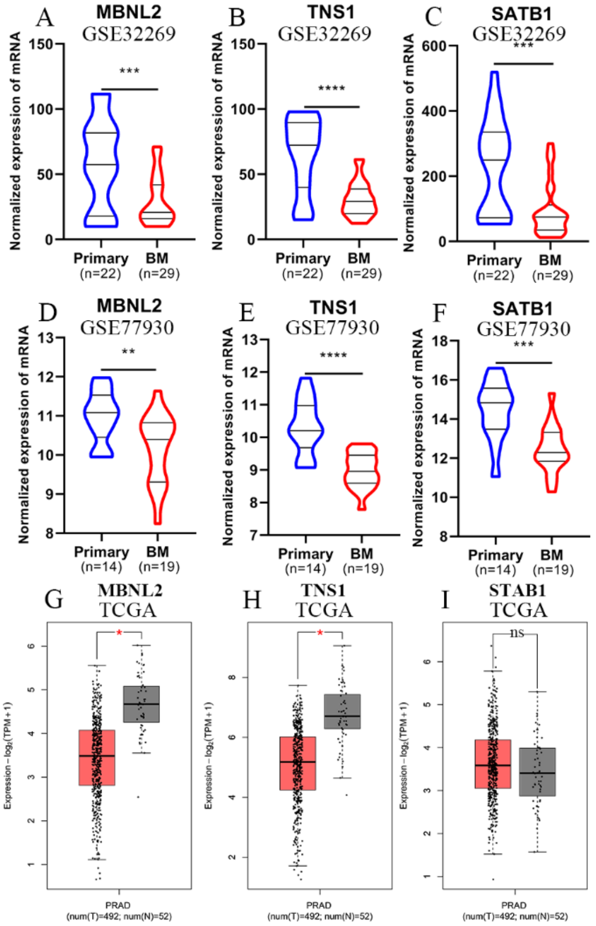

Fig. S4. *MBNL2*, *TNS1*, and *STAB1* expression were elevated in bone metastatic PCa tissues.

(A-C) The expression of *MBNL2*, *TNS1*, and *STAB1* in GEO: GSE32269. (D-F) The expression of *MBNL2*, *TNS1*, and *STAB1* in GEO: GSE77930. (G-I) *MBNL2* and *TNS1* levels were elevated in PCa tissues compared with that in adjacent normal tissues (data from TCGA). ^{ns}, P>0.05; *, P<0.05; **, P<0.01; ***, P<0.001; ****, P<0.0001; Student's

t-test. BM, bone metastatic tissues; N, adjacent normal tissues; ns, no significance; T, tumor tissues.

Table S1. The top 20 hub genes detected using the 12 algorithms in cytoHubba plug-in.

Rank	algorithms											
	MCC	DNMC	MNC	Degree	EPC	BottleNeck	EcCentricity	Closeness	Radiality	Betweenness	Stress	Clustering Coefficient
1	COL1A1	CHRDL1	FN1	FN1	FN1	MMP9	MMP9	FN1	MMP9	MMP9	MMP9	CA1
2	COL1A2	MMP13	COL1A1	COL1A1	COL1A1	COL1A1	ZEB1	MMP9	FN1	FN1	FN1	ZEB1
3	FN1	COL5A3	MMP9	MMP9	COL1A2	TYROBP	VCAM1	COL1A1	COL1A1	TYROBP	CSF1R	SERPINF1
4	COL3A1	PLOD2	COL1A2	COL1A2	COL3A1	FN1	CCL18	COL1A2	COL1A2	CSF1R	TYROBP	DPT
5	POSTN	SERPINF1	COL3A1	COL3A1	MMP9	CSF1R	S100A4	COL3A1	SPP1	COL1A1	COL1A1	OLFML2B
6	SPP1	DPT	SPP1	SPP1	SPP1	CTSZ	S100A8	SPP1	VCAM1	HBB	COL3A1	RNASE1
7	MMP13	COL11A1	POSTN	POSTN	POSTN	POSTN	COL1A2	POSTN	COL3A1	APOE	HBB	OMD
8	MMP9	LAMB1	APOE	VCAN	VCAN	HBB	COL1A1	VCAN	CSF1R	COL3A1	COL1A2	APOC1
9	VCAN	PCOLCE	VCAN	TYROBP	COL4A2	SPP1	HBB	APOE	APOE	SPP1	APOE	DEFA4
10	COL11A1	COL10A1	COL11A1	APOE	COL11A1	THY1	COL3A1	CSF1R	VCAN	SLPI	SPP1	CHRDL1
11	COL4A2	VCAN	CSF1R	COL11A1	SDC1	TGFBI	APOE	TYROBP	SDC1	COL1A2	VCAM1	CNN1
12	PLOD2	MEPE	TYROBP	CSF1R	APOE	VCAM1	PTX3	VCAM1	POSTN	LAPTM5	SDC1	NR4A2
13	LAMB1	IBSP	COL4A2	COL4A2	MMP13	S100A4	TNFAIP6	SDC1	TYROBP	VCAM1	MXRA5	FOSB
14	IBSP	COL4A2	SDC1	SDC1	VCAM1	COL3A1	SPP1	COL4A2	MMP13	SDC1	VCAN	COL5A3
15	S100A4	STAB1	VCAM1	VCAM1	LAMB1	APOE	FN1	MMP13	S100A4	LPL	POSTN	THBS4
16	COL5A3	LGALS1	LAMB1	LAMB1	THY1	LPL	VCAN	IBSP	IBSP	VCAN	SLPI	HBA1
17	COL10A1	TGFBI	IBSP	IBSP	S100A4	COL11A1	CSF1R	COL11A1	COL4A2	POSTN	VSIG4	AHSP
18	APOE	ZEB1	MMP13	MMP13	IBSP	IBSP	TYROBP	THY1	THY1	CD163	CD163	PLOD2
19	MEPE	S100A4	S100A4	THY1	PCOLCE	SLPI	COL4A2	S100A4	CD163	THY1	STAB1	MMP13
20	LGALS1	POSTN	PCOLCE	S100A4	COL10A1	DUSP1	F13A1	LAMB1	F13A1	MXRA5	LAPTM5	STAB1
